# Respiratory bacterial co-infections and their antibiotic resistance pattern in COVID-19 patients at a tertiary care centre in India

**DOI:** 10.1099/acmi.0.000514.v3

**Published:** 2023-06-22

**Authors:** Mitra Kar, Tasneem Siddiqui, Akanksha Dubey, Zia Hashim, Chinmoy Sahu, Ujjala Ghoshal

**Affiliations:** ^1^​ Department of Microbiology, Sanjay Gandhi Postgraduate Institute of Medical Sciences, Lucknow, Uttar Pradesh-226014, India; ^2^​ Department of Pulmonary Medicine, Sanjay Gandhi Postgraduate Institute of Medical Sciences, Lucknow, Uttar Pradesh -226014, India

**Keywords:** COVID-19, bacterial infections, real-time PCR (RT-PCR), intensive care unit (ICU), mechanical ventilation, co-infections

## Abstract

**Introduction.:**

Patients with coronavirus disease-2019 (COVID-19) are prone to develop respiratory bacterial infections irrespective of their need for mechanical ventilatory support.

**Hypothesis/Gap Statement.:**

Information about the incidence of concomitant respiratory bacterial infections in COVID- 19 patients from India is limited.

**Aim.:**

This study aimed to determine the incidence of concomitant respiratory bacterial pathogens and their drug resistance in these patients.

**Methodology.:**

A prospective study was performed by including patients who were admitted to our tertiary care centre from March 2021 to May 2021 to evaluate secondary bacterial respiratory co-infections in patients via real-time PCR (RT-PCR)-confirmed cases of COVID-19 disease caused by SARS CoV-2.

**Results.:**

Sixty-nine culture-positive respiratory samples from patients with COVID-19 were incorporated into this study. The most commonly isolated bacterial microorganisms were *Klebsiella pneumoniae* (23 samples, 33.33 %) and *Acinetobacter baumannii* (15, 21.73 %), followed by *Pseudomonas aeruginosa* (13, 18.84 %). Among the microorganisms isolated, 41 (59.4 %) were multidrug-resistant (MDR) and nine (13 %) were extensively drug-resistant (XDR). Among the Gram-negative bacteria isolated, *K. pneumoniae* showed high drug resistance. Fifty carbapenem-resistant microorganisms were isolated from the patients included in our study. Concerning the hospital stay of the patients enrolled, there was an increased length of intensive care unit stay, which was 22.25±15.42 days among patients needing mechanical ventilation in comparison to 5.39±9.57 days in patients on ambient air or low/high-flow oxygen.

**Conclusion.:**

COVID-19 patients need increased length of hospitalization and have a high incidence of secondary respiratory bacterial infections and high antimicrobial drug resistance.

## Data Summary

No supporting data in publicly available repositories (where available and appropriate) have been added to the main manuscript.

## Introduction

Clinical deterioration occurs due to secondary bacterial infections or bacterial co-infections that are known complications of viral respiratory infections. In previously conducted studies during the influenza pandemic and seasonal influenza, bacterial co-infection-related morbidity and mortality were increased in patients with bacterial co-infections [1, 2]. Coronavirus disease-2019 (COVID-19) was first identified in early December 2019 and since then has been an ongoing pandemic. Severe acute respiratory syndrome coronavirus 2 (SARS-CoV-2) is an enveloped RNA beta-coronavirus that is the causative agent of COVID-19. Both SARS-CoV-2 and severe acute respiratory syndrome coronavirus (SARS-CoV) belong to the subgenus *Sarbecovirus* of the family *Coronaviridae* [3]. Other coronaviruses, such as SARS-CoV and Middle East respiratory syndrome coronavirus (MERS-CoV) are reported to be associated with bacterial and fungal co-infections [4–7].

COVID-19 is associated with an increased the length of hospital stay and incidence of nosocomial infections [8]. Infections acquired during hospital stay after 48–72 h of admission are described as nosocomial infections, and their mechanism of spread is mainly from one person to person, and thorough contact with medical devices and instruments [9]. Among the bacterial co-infections that are nosocomially acquired, common causative agents are *Staphylococcus* spp. and *Enterococcus* spp. among Gram-positive cocci, and *Klebsiella pneumoniae *, *Enterobacter* spp*., Escherichia coli, Acinetobacter* spp*.* and *Pseudomonas* spp. among Gram-negative bacilli [10]. Superinfections in combination with viral respiratory infections are commonly caused by the above-mentioned microorganisms in patients hospitalized or maintained on mechanical ventilation for a prolonged period [11]. Underlying comorbidities are also a risk factor for co-infections among people of all age groups [12, 13].

The risk of ventilator-associated bacterial pneumonia in patients with COVID-19 has been studied by Póvoa *et al*., and only a few other retrospective reports of such co-infections have been published [14–16]. The present study aims to report the common microorganisms causing respiratory bacterial co-infections in COVID-19 patients. We also reviewed drug resistance among microorganisms causing bacterial co-infection in COVID-19 patients while noting the causative microorganisms and risk factors leading to a multidrug-resistant (MDR) or xtensively drug-resistant (XDR) infection.

## Methods

### Study design and setting

We performed a prospective observational study from March 2021 to May 2021, during the second wave of COVID-19 to assess secondary bacterial respiratory infections among patients with COVID-19 in the Bacteriology section at a tertiary care centre in northern India. Ethical approval for this study was obtained from the ethical committee of the institution (IEC code: 2021–89-EMP-EXP-37 PGI/BE/1218/2021 dated 17 April 2021). All procedures followed the ethical standards of the responsible committee on human experimentation (institutional or regional) and with the Helsinki Declaration of 1975.

### Clinical specimens and laboratory examination

We included all patients with secondary bacterial respiratory infections during or after they tested positive for COVID-19 infection and were admitted to the general ward as well as the intensive care unit (ICU) of the tertiary care centre. We also differentiated between patients who had to undergo mechanical ventilation and those who were on ambient air or low/high-flow oxygen by closely monitoring the period of hospitalization to the intensive care unit, length of intubation and death rate among these patients. The risk factors related to 30 day mortality of patients with concomitant respiratory bacterial co-infections were also observed over the study duration. In all cases, a positive real-time PCR (RT-PCR) report was required for diagnosing COVID-19 and admission to the tertiary care centre.

**Fig. 1.**
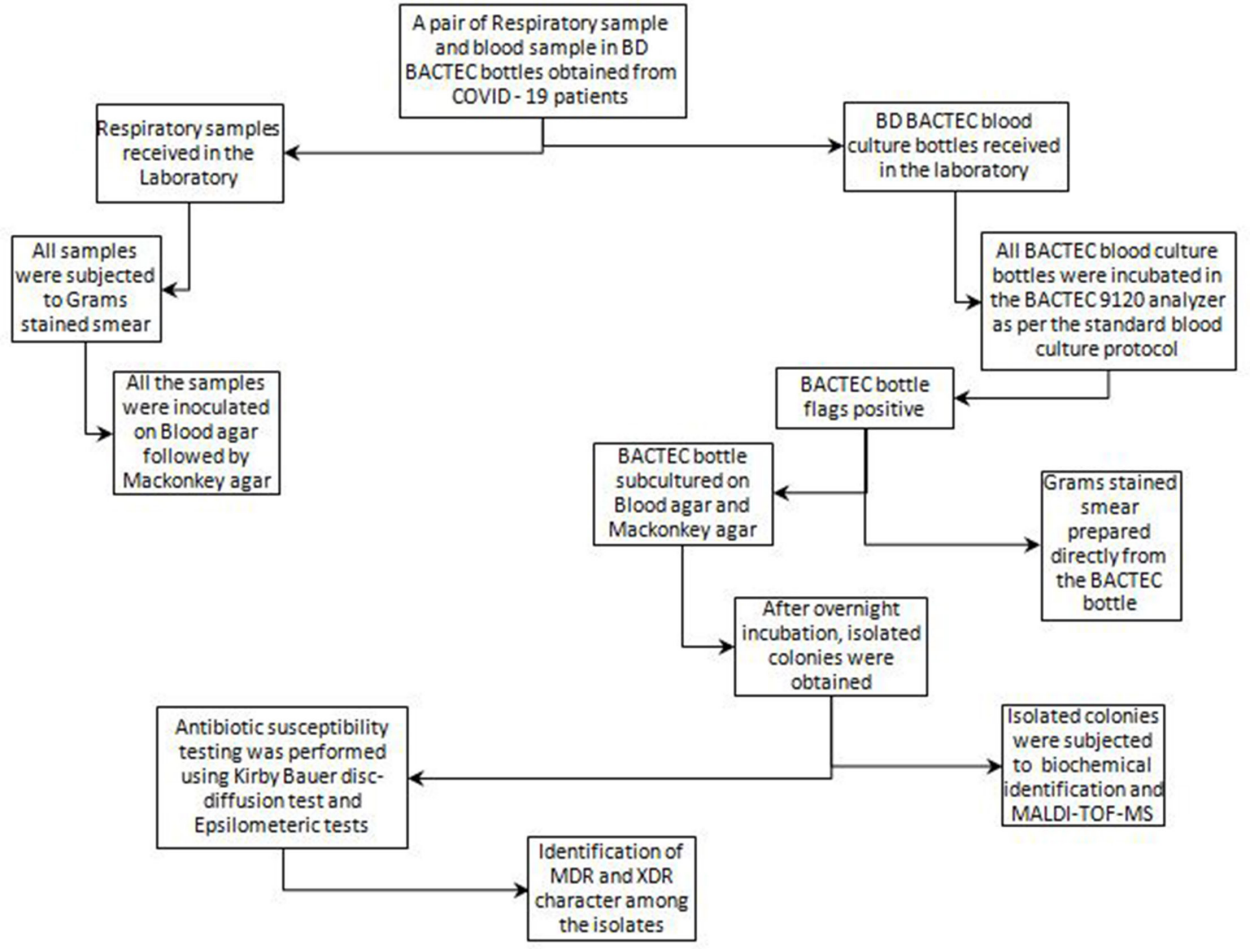
Flowchart of tests performed on the samples acquired in our laboratory.

A pair of respiratory and blood samples was obtained at admission from all patients. The respiratory samples included in our study were bronchoalveolar lavage, tracheal aspirates, endotracheal aspirates, throat swabs and sputum samples. Fig. 1 describes the flow of tests performed on the samples acquired in our laboratory. All respiratory samples were subjected to Gram staining and inoculated on Mackonkey and blood agar. All inoculated Mackonkey agar plates were incubated at 37 °C overnight in an aerobic incubator while all inoculated blood agar plates were incubated at 37 °C overnight in a 5–10 % CO_2_ incubator. All blood culture samples were sent in BD BACTEC bottles and incubated in a BACTEC 9120 analyser until flagged as positive or removed after 5 days of incubation as per our standard blood culture protocol. All positively flagged BACTEC bottles were subjected to Gram staining and subcultured on blood agar and Mackonkey agar. Identification of each isolate was primarily performed using biochemicals and these identifications were confirmed by matrix-assisted laser desorption/ionization – time of flight MS (MALDI-TOF-MS). The taxonomic affiliations of each bacterial isolate could not be confirmed using 16S rRNA gene sequencing due to lack of funds.

Antibiotic sensitivity testing was performed for all microorganisms isolated from respiratory and blood samples using the Kirby Bauer disc-diffusion method and Epsilometeric-test method in accordance with CLSI guidelines [17]. A broth microdilution test could not be performed for the isolates due to unavailability of the powder form of the antibiotics and a lack of funds for their purchase. Microbiological characteristics and drug resistance patterns were extensively analysed for respiratory samples included in our study. Isolation of MDR and XDR microorganisms and their risk factors were also established; MDR microorganisms were identified by resistance to one or more drugs of three different classes of antibiotics, which also included *Enterobacteriaceae* that produce extended-spectrum beta lactamases (ESBLs) and are carbapenemase-resistant, XDR microorganisms were identified as sensitive to less than one antibiotic group such as isolates from respiratory samples that were only susceptible to either colistin, minocycline or ELORES (ceftriaxzone – EDTA), *Pseudomonas aeruginosa* resistant to three antibiotic groups, vancomycin-resistant *Enterococcus* and methicillin-resistant *Staphylococcus aureus* [18, 19]. We could not execute any molecular methods for demonstration of gene-mediated or plasmid-mediated antibiotic resistance due to a lack of funds.

The D-dimer, serum ferritin, procalcitonin, positive fibrinogen degradation product, total leucocyte count and bacteraemia parameters of these patients were also compiled from the hospital information system. In this study, the demographic characteristics observed included age, gender and comorbidities, which include hypertension, diabetes, chronic respiratory disease, chronic renal disease, neoplasm, immunosuppression and postoperative conditions and were extracted from the electronic records of the patients. Immunosuppression was defined in patients receiving treatment with immunosuppressant drugs, haematological malignancies, transplant recipients and with uncontrolled diabetes.

### Statistical analysis

Statistical analysis was done by observing frequencies. The mean and standard deviation are used to express quantitative variables. In the analysis of risk factors for MDR, the comparison between groups for categorical variables was estimated by using χ^2^ tests. The results are presented as 95 % confidence intervals (CI). Statistical analysis was performed using the software program IBM SPSS Statistics version 20.0 (SPSS), with *P*<0.05 considered statistically significant.

## Results

Our study included 69 patients with COVID-19 caused by SARS-CoV-2. Thirty-six patients (52.17 %) needed mechanical ventilation, and 33 (47.83 %) patients were on ambient air or low/high-flow oxygen. All the patients included in this study were diagnosed with a secondary bacterial respiratory infection based on microscopic examination and routine aerobic bacterial culture. Of the 69 patients, the patients included in our study had a mean±sd age of 52.03±17.08 years (range 12–86 years), 52 (75.4 %) were male and 17 (24.6 %) were female.

Of the 69 patients included in our study, 67 (97.1 %) had comorbidities such as hypertension, chronic respiratory disease, diabetes mellitus, chronic renal disease, neoplasm, immunosuppressant use and postoperative complications. At the end of the study, 27 patients (39.1 %) died due to respiratory infections with a mean age of 56.07±16.67 years. Hypertension and chronic respiratory disease were the common comorbidities in those who were enrolled in the study, accounting for 33 (47.82 %) and 37 (53.62 %) patients, respectively. Chronic respiratory disease and neoplastic diseases showed a significant association with COVID-19-positive patients who need mechanical ventilation. Concerning the hospital stay of the patients enrolled, there was an increased length of ICU stay, which was 22.25±15.42 days among patients needing mechanical ventilation in comparison to 5.39±9.57 days of ICU stay in patients on ambient air or low/high-flow oxygen. In our study, the duration of stay in the ICU was significantly associated with COVID-19-positive patients who needed mechanical ventilation. Table 1 demonstrates the above-discussed descriptive demographics.

**Table 1.** Descriptive analysis of demographic characteristics and risk factors in patients with secondary respiratory tract infection among COVID-19-positive patients (*N*=69)

	Total (*N*=69)	With MV (*n*=36)	Without MV (*n*=33)	*P*
**Demographics**				
Age, years, mean (sd)	52.03±17.08	54.44±15.35	49.39±18.67	0.222
Gender (male), *n* (%)	52 (75.4 %)	25 (69.4 %)	27 (81.8 %)	0.233
**Comorbidities**				
Diabetes mellitus, *n* (%)	22 (31.9 %)	9 (25 %)	13 (39.39 %)	0.200
Hypertension, *n* (%)	33 (47.82 %)	17 (47.22 %)	16 (48.48 %)	0.916
Chronic respiratory disease, *n* (%)	37 (53.62 %)	26 (72.22 %)	11 (33.33 %)	**<0.001**
Chronic renal disease, *n* (%)	18 (26.08 %)	10 (27.77 %)	8 (24.24 %)	0.738
Neoplasm, *n* (%)	7 (10.1 %)	1 (2.77 %)	6 (18.18 %)	**0.049**
Immunosuppressant use, *n* (%)	19 (27.53 %)	7 (19.44 %)	12 (36.36 %)	0.116
Postoperativ, *n* (%), %	12 (17.39 %)	4 (11.11 %)	8 (24.24 %)	0.151
**History of hospital stay**				
ICU hospitalization length (days), mean (sd)	14.19±15.41	22.25±15.42	5.39±9.57	**<0.001**
Intubation length (days), mean (sd)	12.28±9.35	12.28±9.35	–	**<0.001**
Death, *n* (%)	27 (39.13 %)	24 (66.67 %)	3 (9.09 %)	**<0.001**

An independent samples *t*-test was used to compare means; a chi-square test/Fisher's exact test was used to compare the proportions with and without MV. Significant *P-v*alues (<0.05) are in bold type.

MV, mechanical ventilation.

**Fig. 2.**
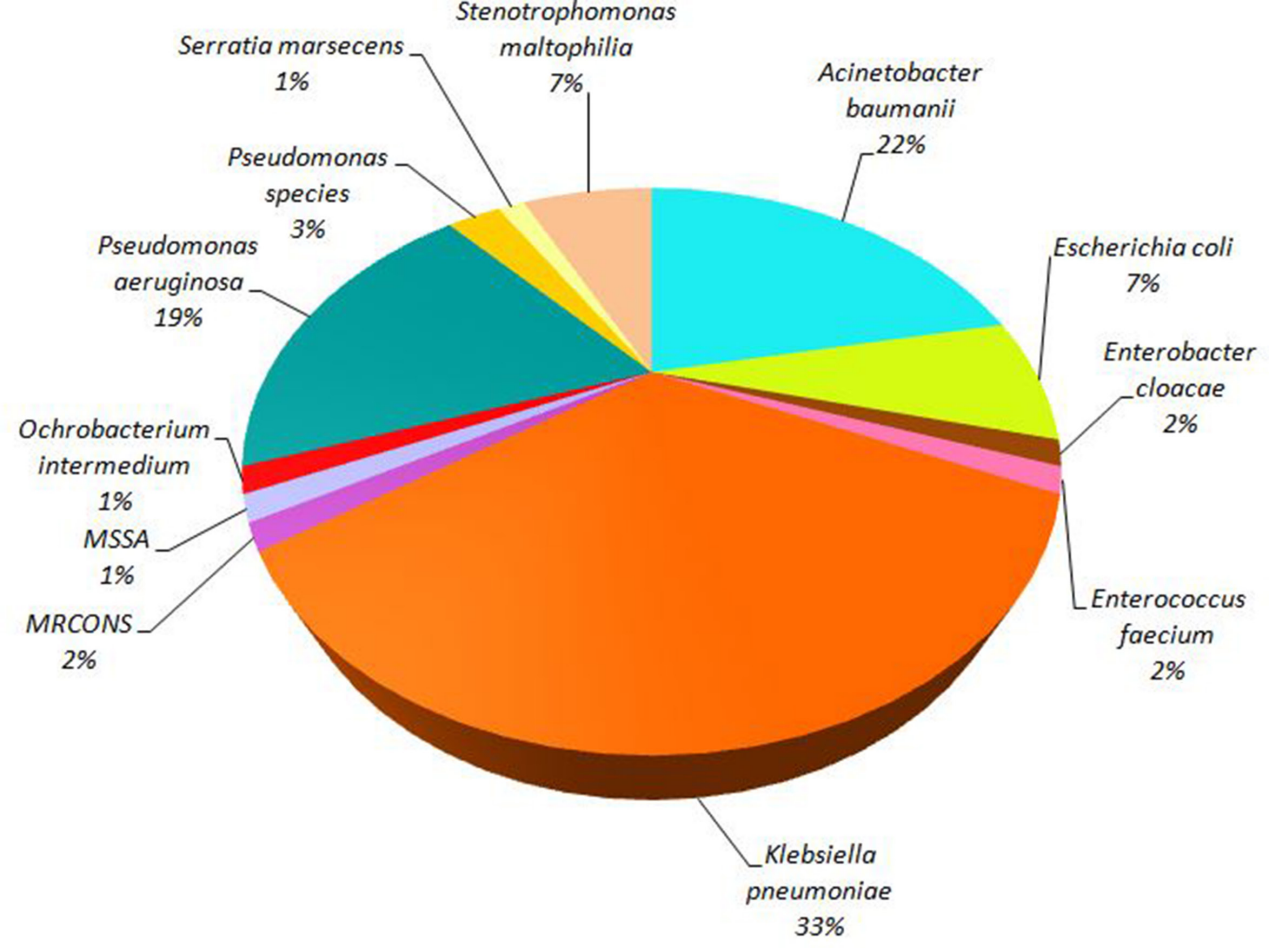
Distribution of bacterial pathogens in respiratory co-infections in COVID-19 patients (*N*=69). MSSA, methicillin-sensitive *Staphylococcus aureus *. MRCONS, Methicillin resistant coagulase negative *Staphylococcus *.

Among patients requiring mechanical ventilation, *Acinetobacter baumannii* and *Pseudomonas aeruginosa* were the most commonly isolated microorganisms, 11 (11/36, 30.55 %) each, followed by *Klebsiella pneumoniae *, 8 (8/36, 22.22 %). Among the patients who were being managed on ambient air or low/high-flow oxygen, 15 (15/33, 45.45 %) developed a secondary respiratory infection of *K. pneumoniae *. The most commonly isolated bacterial microorganisms among all the respiratory samples sent to the Bacteriology Section of the Microbiology department were *K. pneumoniae* (23/69, 33.33 %) and *Acinetobacter baumannii* (15/69, 21.73 %), followed by *Pseudomonas aeruginosa* (13/69, 18.84 %), as shown in Fig. 2.

**Fig. 3.**
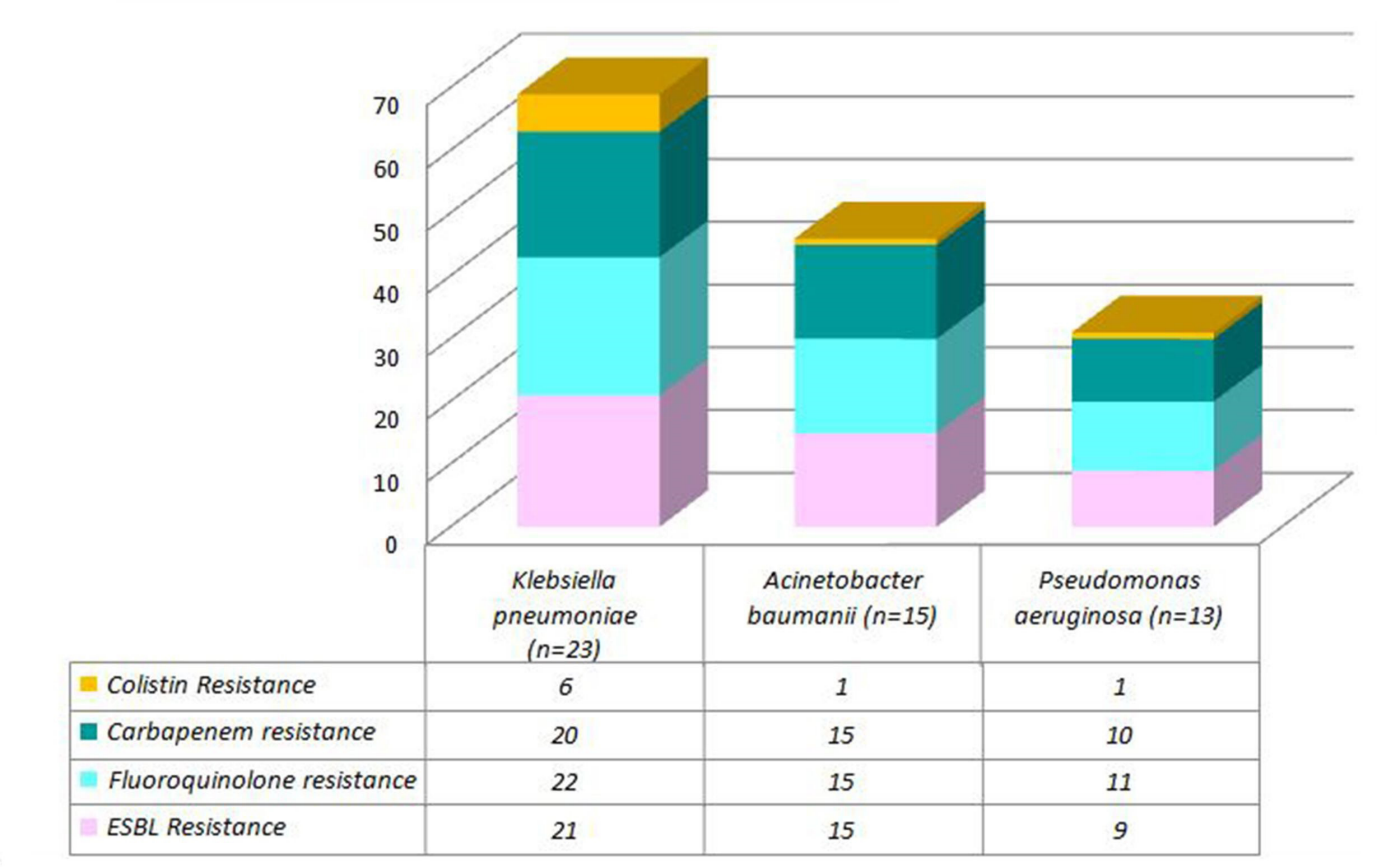
Antibiotic resistance to the major groups of antibiotics among the most resistant microorganisms isolated from the respiratory samples in COVID-19 patients (*N*=51).

Antimicrobial susceptibility testing showed that the isolates of *K. pneumoniae *, *A. baumannii* and *P. aeruginosa* were highly resistant to all the antibiotics, except for colistin, where only 11.59 % (8/69) of isolates showed resistance, as represented in Fig. 3. The resistance pattern of *K. pneumoniae* was greater among mechanically ventilated patients in comparison to isolates from patients on ambient air or low/high-flow oxygen, whereas, in the case of *A. baumannii *, the resistance pattern of the isolates did not vary between mechanically or non-mechanically ventilated patients. Among the Gram-positive microorganisms, only one *Staphylococcus aureus* isolate was observed among the various respiratory samples but was found to be methicillin-sensitive *S. aureus* (MSSA) and resistant to levofloxacin and erythromycin only. The patient from whom the MSSA strain was isolated was discharged after 20 days of stay in the general COVID-19 ward of this facility. Another Gram-positive isolate, of *Enterococcus faecium,* was obtained from a patient admitted to the COVID ICU for 41 days and intubated for mechanical ventilation for 13 days. The above isolate was XDR and resistant to both vancomycin and linezolid, and the patient died on the 42nd day of the COVID ICU stay.

**Fig. 4.**
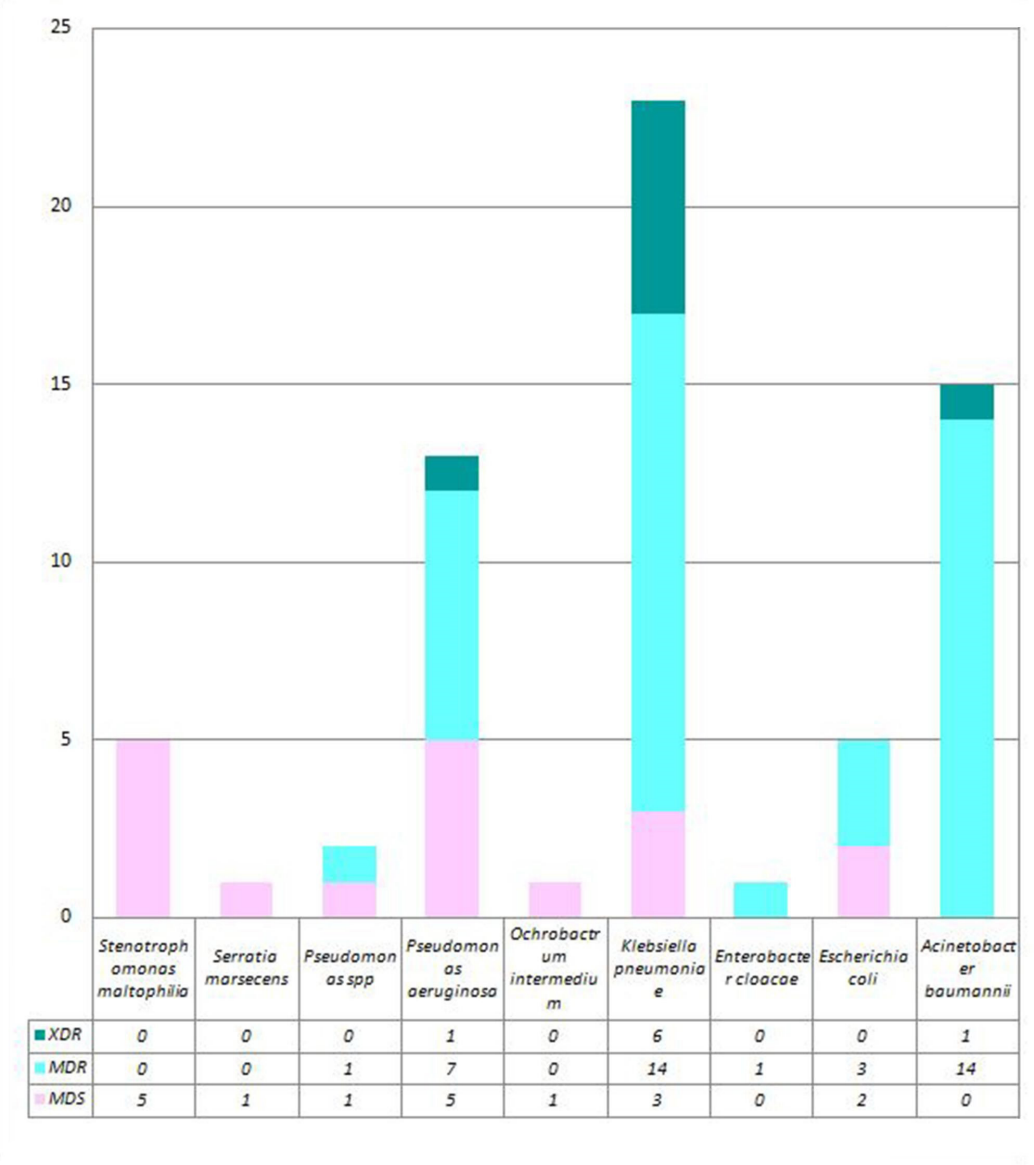
Organism-wise distribution of multidrug-sensitive (MDS), multidrug-resistant (MDR) and extensively drug-resistant bacteria among Gram negative bacilli isolated from our study cohort (*N*=69).

Among the microorganisms isolated from the respiratory samples, 41 (59.4 %) were MDR and nine (13 %) were XDR, as depicted in Fig. 4. The total number of *Enterobacteriaceae* in the study was 30 (30/69, 43.48 %). There were 24 (24/30, 80 %) MDR *Enterobacteriaceae* in this study, of which eight (8/8, 100 %) showed resistance to third-generation cephalosporins in those patients who needed mechanical ventilation and 19 (19/22, 86.36 %) in patients who were managed on ambient air or low/high-flow oxygen. A total of 23 isolates of *K. pneumoniae* were isolated from the samples included in our study, of which eight patients were on mechanical ventilation and 15 were on ambient air or low/high-flow oxygen. *K. pneumoniae* showed higher drug resistance: eight (8/8, 100 %) of the isolates were ESBL-producing *K. pneumoniae* in patients needing mechanical ventilation and 13 (13/15, 86.67 %) in patients who were managed on ambient air or low/high-flow oxygen. There were 50 carbapenem-resistant organisms isolated from patients included in the study (77.78 and 11.11 % in patients who were managed on mechanical ventilation and ambient air or low/high-flow oxygen respectively), of which 90.90% of carbapenem-resistant *P. aeruginosa* were isolated from patients on mechanical ventilation was and 9.1 % from those on ambient air or low/high-flow oxygen. *P. aeruginosa* showed complete resistance to piperacillin-tazobactam in patients on mechanical ventilation, while those isolates observed from patients without mechanical ventilation were all susceptible to piperacillin-tazobactam. All 15 isolates of *A. baumannii* in our study were carbapenem-resistant; of these isolates, 11 (11/15, 73.33 %) were from paients tients on mechanical ventilation, while four (4/15, 26.67 %) were from patients on ambient air or low/high-flow oxygen support. Isolates were highly resistant to quinolones in 57 patients (82.6 %) among the COVID-19-positive patients admitted to this tertiary care centre; 31 (31/36, 86.11 %) and 26 (26/33, 78.78 %) isolates were quinolone-resistant among patients managed on mechanical ventilation and ambient air respectively. Aminoglycoside resistance among *P. aeruginosa A. baumannii* and *K. pneumoniae* was observed in 66.67, 100 and 82.6 % of isolates, respectively. Finally, a vancomycin-resistant *Enterococcus faecium* (VRE) isolate was treated with daptomycin in a patient who was on mechanical ventilation and died on the 42nd day of ICU stay.

MDR and XDR microorganisms were collectively observed in 28 (28/36, 77.78 %) patients who needed mechanical ventilation and in 22 (22/33, 66.67 %) patients on low/high-flow oxygen. The incidence of MDR organisms was higher among patients with comorbidities such as chronic respiratory disease and hypertension. Table 2 demonstrates the average length of ICU stay in patients with MDR and XDR respiratory infections, which was 15.07±14.42 and 17.33±15.93 days, respectively. A mortality rate of 55.5 % (5/9) was observed in patients with XDR secondary bacterial respiratory infections compared to 39.02 % (16/41) in patients contracting MDR secondary bacterial respiratory infections, but these results were not statistically significant. As shown in Table 3, total leucocyte counts were higher in patients who were placed on mechanical ventilation than in those who were on ambient air or low/high-flow oxygen. Of 69 positive respiratory samples, 18 (18/69, 26.08 %) showed concomitant bloodstream infections, and of these 18 samples, 12 (12/18, 66.67 %) were from mechanically ventilated patients. Among the microorganisms isolated from bloodstream infections in our study cohort, *P. aeruginosa* (4/18, 22.22 %) and methicillin-resistant *S. aureus* (MRSA) (4/18, 22.22 %) were identified as the most common isolates. Of these isolates, only one isolate among *P. aeruginosa* (1/4, 25.0 %) was sensitive to only one class of antibiotics making it XDR and one isolates among MRSA (1/4, 25.0 %) was found to be MDR with susceptibility only to vancomycin and teicoplanin, and was resistant to fluoroquinolones, ESBL antibiotics and penicillins.

**Table 2.** Clinical factors for the isolation of drug-resistant microorganisms in COVID-19 patients with a secondary bacterial respiratory infection (*N*=50)

	Total drug resistance (*N*=50)	Incidence of MDR (*n*=41)	Incidence of XDR (*n*=9)	*P*	95 % CI
**Demographics**					
Age, years, mean (sd)	52.78±17.59	52.44±17.56	54.33±18.73	0.773	47.78–57.78
Gender, male, *n* (%)	42 (84 %)	33 (80.48 %)	9 (100 %)	0.148	1.05–1.27
**Comorbidities**					
Diabetes mellitus, *n* (%)	17 (34 %)	14 (34.14 %)	3 (33.33 %)	0.963	1.52–1.80
Hypertension, *n* (%)	25 (50 %)	22 (53.65 %)	3 (33.33 %)	0.269	1.36–1.64
Chronic respiratory disease, *n* (%)	27 (54 %)	23 (56.09 %)	4 (44.44 %)	0.525	1.32–1.60
Chronic renal disease, *n* (%)	13 (26 %)	9 (21.95 %)	4 (44.44 %)	0.164	1.61–1.87
Neoplasm, *n* (%)	4 (8 %)	3 (7.31 %)	1 (11.11 %)	0.527	1.84–2.00
Immunosuppressant use, *n* (%)	14 (28 %)	11 (26.82 %)	3 (33.33 %)	0.694	1.71–1.93
Postoperative condition, *n* (%)	9 (18 %)	7 (17.07 %)	2 (22.22 %)	0.716	1.59–1.85
**History of hospital stay**					
ICU hospitalization length (days), mean (sd)	15.48±14.56	15.07±14.42	17.33±15.93	0.678	11.34–19.62
Intubation length (days), mean (sd)	7.28±9.93	7.48±10.50	6.33±7.12	0.446	4.46–10.10
Died, *n* (%)	21 (42 %)	16 (39.02 %)	5 (55.55 %)	0.363	0.44–0.72

A *P*-value of <0.05 is significant. MDR=multidrug resistant, XDR=extensively drug-resistant.

**Table 3.** Laboratory characteristics of COVID-19 patients with secondary respiratory tract infections, with and without mechanical ventilation (*N*=69)

Laboratory parameters	Total (*N*=69)	With mechanical ventilation (*n*=36)	Without mechanical ventilation (*n*=33)	*P*
D-dimer (μg/ml), mean (sd)	3.32±2.56	3.36±2.93	3.28±2.08	0.897
Serum ferritin (ng/ml), mean (sd)	2070.54±2610.97	2232.26±3378.99	1890.15±1368.63	0.589
Pro-calcitonin assay, mean (ng/ml) (sd)	5.05±8.34	4.67±7.73	5.51±9.12	0.680
Positive fibrinogen degradation product, *n* (%)	49 (71.01 %)	28 (77.77 %)	21 (63.63 %)	0.420
Total leucocyte count (cells/ cubic mm), mean (sd)	14286.81±11 792.114	16630±14 490.89	11730±7273.4	0.0847
Bacteraemia, *n* (%)	18 (26.08 %)	12 (33.33 %)	6 (18.18 %)	0.152

A *P*-value of <0.05 is significant.

The risk factors related to 30 day mortality of patients with respiratory bacterial coinfection with COVID-19 are listed in Table 4. The overall mortality recorded in our study was 39.10 % (27/69) and 26.10 % (18/69) was associated with bacteraemia. Among the risk factors discussed, mechanical ventilation, length of hospital stay and total leucocyte count were statistically significant risk factors among those who died in comparison to those who survived in our study. The value of procalcitonin was higher in patients who died as a result of their infections and thus procalcitonin was a valuable marker for diagnosis of sepsis and impending mortality.

**Table 4.** Risk factors related to 30 day mortality of patients with concomitant bacterial respiratory co-infections with COVID-19 (*N*=69)

Parameters	Total (*n*=69)	Survived (*n*=42)	Died (*n*=27)	*P*
**Age**				
>65 years, *n* (%)	24 (34.78 %)	6 (14.71 %)	18 (66.67 %)	0.061
≤65 years, *n* (%)	45 (65.22 %)	36 (85.71 %)	9 (33.33 %)	0.061
**Gender**				
Male, *n* (%)	52 (75.36 %)	34 (80.95 %)	18 (66.67 %)	0.179
Female, *n* (%)	17 (24.64 %)	8 (19.05 %)	9 (33.33 %)	0.179
**Risk factors for bacterial respiratory co-infections in COVID-19 patients**				
Mechanical ventilation/intubation, *n* (%)	36 (52.17 %)	12 (28.57 %)	24 (88.89 %)	<0.001*
Neoplasm, *n* (%)	7(10.14 %)	4(9.52 %)	3(11.11 %)	0.831
Hypertension, *n* (%)	33 (47.83 %)	19 (45.24 %)	14 (51.85 %)	0.591
Immunosuppression, *n* (%)	19 (27.54 %)	14 (33.33 %)	5 (18.52 %)	0.179
Post-operative conditions, *n* (%)	12 (17.39 %)	8 (19.05 %)	4 (14.81 %)	0.651
Chronic respiratory disease, *n* (%)	37 (53.62 %)	20 (47/62 %)	17 (62.96 %)	0.212
Diabetes mellitus, *n* (%)	22 (31.88 %)	14 (33.33 %)	8 (29.63 %)	0.747
Chronic renal disease, *n* (%)	18 (26.09 %)	9 (21.43 %)	9 (33.33 %)	0.272
**Other parameters**				
Length of hospital stay (days), mean±sd (range)	14.19±15.41	10.45±12.34	20.00±17.99	0.0109*
Procalcitonin (normal range <0.5 ng/ml), mean±sd	5.06±8.34	4.30±8.28	6.14±8.45	0.375
Leucocytes (normal range 4000–10000 cells/ cubic mm), mean±sd	14286.81±11 792.11	12231.03±6679.11	17840.00±16 603.39	0.05*

*A *P*-value of ≤0.05 is statistically significant.

**Fig. 5.**
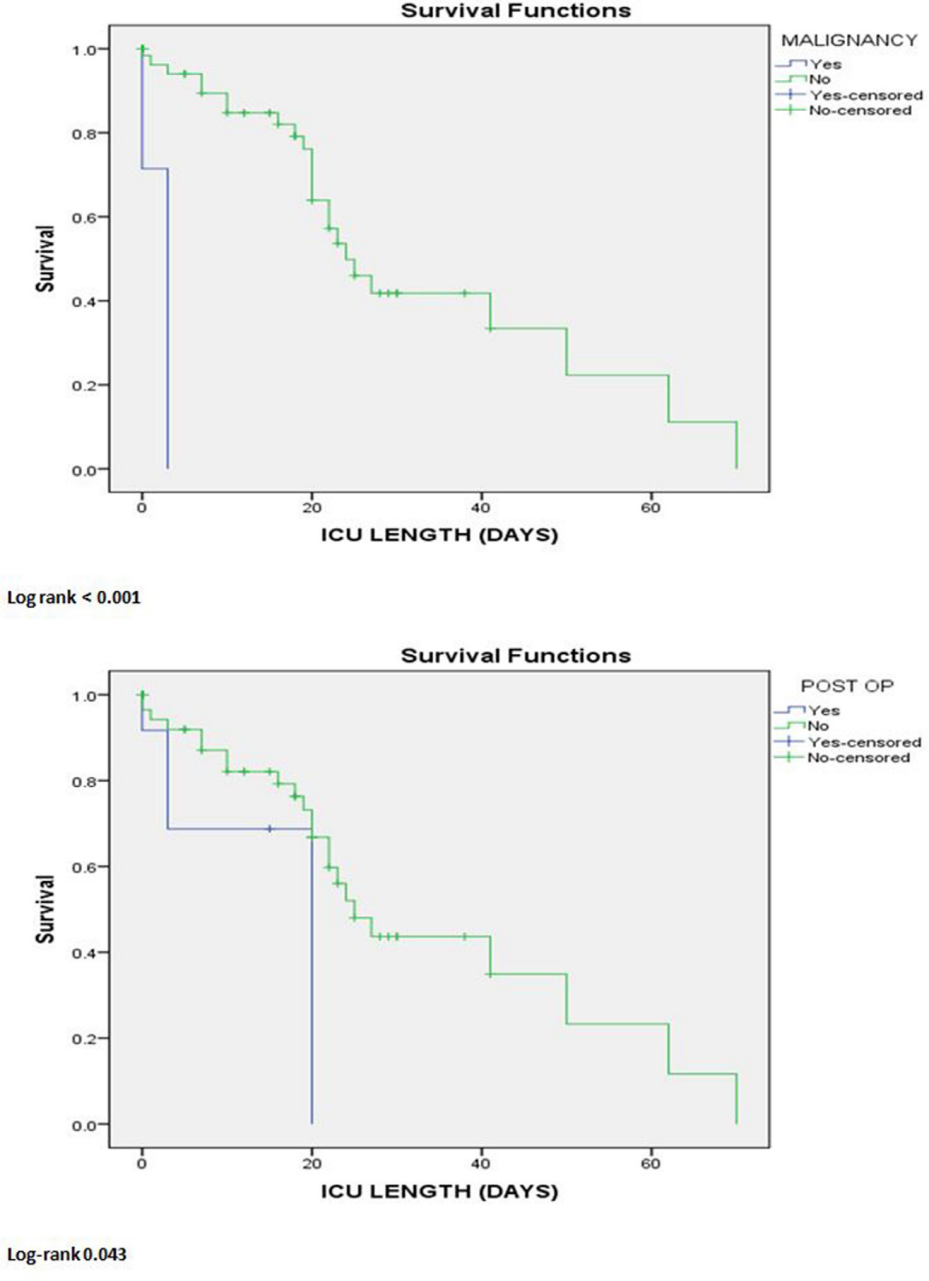
Kaplan–Meier survival analysis for univariate analysis between patients with and without mechanical ventilation and postoperative conditions (*N*=69).

According to Kaplan–Meier survival analysis for univariate analysis of patients with secondary bacterial respiratory infections with COVID-19, as shown in Fig. 5, those not having any malignancy and those who had not undergone recent operative procedures were significantly associated with a better survival rate among. Further, multivariate Cox regression analysis showed that the absence of malignancy was associated with a better survival rate among COVID-19 patients with secondary respiratory bacterial infections.

## Discussion

Bilateral pneumonia is the most common complication in COVID-19 infections, which may progress to acute respiratory distress syndrome (ARDS), and those with serious illness are more prone to develop complications [20]. Many patients with COVID-19 were admitted to a COVID treatment facility leading to admission to an ICU, and their ventilation was maintained using a mechanical ventilator. Life-saving, non-physiological and invasive intervention used among COVID-19 patients is positive pressure ventilation [21]. A correlation between bacterial co-infection and SARS-CoV-2 has been in previous studies [22]. A >2-fold increased risk of death was seen in cases of bacterial and fungal co-infections [23], which confirms the interaction among bacterial isolates and SARS-CoV-2. The epithelial damage and delayed ciliary clearance caused by SARS-CoV-2 viral infection facilitate viral and bacterial co-infection. The immune system dysfunction caused by the SARS-CoV-2 is by its ability to damage lymphocytes, particularly B cells, T cells and natural killer cells, may be the cause of co-infection [24].

In our study, 69 patients were reported to have secondary bacterial respiratory coinfections, and 27 (27/69, 39.1 %) of these patients died. A similar study with 19 mechanically ventilated COVID-19-positive patients was performed in Iran, who all were reported to have developed secondary bacterial pneumonia during their hospital stay and all except one died [25]. In studies conducted in China and the UK, secondary bacterial infections were reported in 13.9 and 6.1% respectively [26, 27]. The results of the studies mentioned above are different due to differences in the care provided to the admitted patients and the rate of infections acquired during ICU stay, admission/discharge criteria, infection control measures practised by the hospital staff, and the workload/nurse ratio.

In this study, the most common samples obtained in the laboratory were sputum (33/69, 47.83 %) and endotracheal aspirate samples (22/69, 31.88 %). Bronchoalveolar lavage sampling in COVID-19 positive patients was not advised. Although this procedure has a better yield for identifying the causative pathogens, being an aerosol-generating procedure it was contraindicated in patients with COVID-19 to reduce the risk of transmission [28, 29].

Fifty (50/69, 72.46 %) out of the 69 bacterial isolates identified from the respiratory samples obtained from the COVID-19-positive patients were drug-resistant. Of the 50 drug-resistant isolates, 41 (41/50, 82 %) were MDR and nine (9/50, 18 %) were XDR. Similar observations were made in a study conducted in Egypt, where almost all the bacterial isolates identified from the respiratory samples of COVID-19-positive patients were MDR due to the administration of scheduled antibiotics as mentioned in the COVID-19 protocol that was able to eliminate the susceptible pathogens, leading to an increase in survival of resistant pathogens [30].

The commonest microorganisms obtained from the respiratory samples were the poly drug-resistant (PDR) *K. pneumoniae* (21, 91.30 %) and *A. baumannii* (15, 100 %), similar to a study by Maewed *et al.* [30]. The existence of hypervirulent strains of both the above mentioned microorganisms have been reported by numerous studies [31, 32]. The other causative agents of secondary bacterial pulmonary infections in our study included *Pseudomonas aeruginosa* and *Escherichia coli* followed by *Stenotrophomonas maltophilia,* MSSA and *Serratia marcescens *. The most commonly isolated pathogens from the non-COVID-19 ICU patients in countries including India, Egypt, Iran and China are *K. pneumoniae* and *A. baumannii* [33].

Among the patients included in our study, comorbidities such as chronic obstructive pulmonary disease (COPD) (37/69, 53.62 %) and neoplasm (7/69, 10.1 %) were statistically significant in patients on mechanical ventilation and those managed on ambient air or low/high-flow oxygen. Several studies have demonstrated that COPD is associated with poor disease progression, and a meta-analysis of several studies in China revealed a 4-fold increase in mortality in patients who develop COVID-19 with pre-existing COPD [34].

In our study, among the patients admitted to the ICU, the mean length of ICU stay was higher, 14.19±15.41 days, than in a study by Zhou *et al.*, where the mean length of ICU stay was 8.0 days (4.0–12.0). On admission, no bacterial pathogen was identified in their respiratory samples [35]. The bacterial pulmonary co-infections prolonged the length of hospital stay in the patients. It was reported in a study that patients with pandemic 2009 influenza A (H1N1) virus infection who were co-infected with respiratory bacterial pathogens had an increased length of ICU stay equal to of ≥3 days than those with no co-infection [36].

The mortality rate among patients in our study who were concomitantly infected with SARS-CoV-2 and bacterial microorganisms infecting the respiratory system was 39.1 % (27/69) similar to a study by Toufen *et al.* on ICU patients in Brazil, where the most common secondary infection was respiratory bacterial infection accounting for a mortality rate of 34.7 %, while the total rate of mortality was 28.8 % [37]. The mortality rate of ventilator-associated pneumonia (VAP) was reported to be higher in ICU patients, varying from 20 to 50% when the respiratory infection was caused by a high-risk pathogen, as observed by Chastre *et al.* [38].

This study had some limitations. First, the study was performed at a single centre and thus our findings cannot be applied to the whole population of a geographical area. Second, this study focused only on bacterial respiratory infections, excluding all other infections. Third, there was a lack of knowledge about the practice followed by the clinicians in starting the empirical antibiotic therapy. Lastly, all bacterial isolates were taken into account as all patients included in our study were febrile and were symptomatic of respiratory infections.

## Conclusion

Patients admitted to COVID-19 facilities with or without comorbidities are prone to secondary bacterial pulmonary infections as the virus makes the respiratory system susceptible to infection by causing direct epithelial injury and delayed ciliary clearance. The secondary bacterial pulmonary infections related to patients on mechanical ventilation and those managed on ambient air are mostly caused by microorganisms such as *K. pneumonia, A. baumannii, Serratia marcescens, Enterobacter cloacae, P. aeruginosa, Stenotrophomonas maltophilia,* MSSA and vancomycin-resistant *Enterococcus faecium* when samples were collected by maintaining all aseptic precautions. Microorganisms isolated from the samples showed high antimicrobial resistance, and their incidence should be curbed by adhering to hospital infection control practices to halt the spread of nosocomial infections among patients admitted to the COVID-19 facilities who need prolonged admission in the general ward or stay in the ICU.
